# Editorial: Digital linguistic biomarkers: beyond paper and pencil tests, volume II

**DOI:** 10.3389/fpsyg.2023.1358852

**Published:** 2024-01-10

**Authors:** Jon Andoni Duñabeitia, Dimitrios Kokkinakis, Gloria Gagliardi

**Affiliations:** ^1^Centro de Investigación Nebrija en Cognición, Nebrija University, Madrid, Spain; ^2^Department of Languages and Culture, UiT The Arctic University of Norway, Tromsø, Norway; ^3^Department of Swedish, Multilingualism, Language Technology, University of Gothenburg, Gothenburg, Sweden; ^4^Centre for Ageing and Health (AgeCap), University of Gothenburg, Gothenburg, Sweden; ^5^Department of Classical Philology and Italian Studies, University of Bologna, Bologna, Italy

**Keywords:** linguistic biomarkers, cognitive evaluation, aging, paper and pencil, cognitive test

As the co-editors of the second edition of “*Digital linguistic biomarkers: beyond paper and pencil tests*,” we are pleased to present this Research Topic of cutting-edge research articles that continue to explore the exciting intersection of linguistics, technology, and cognitive science. Building upon the success of our first volume in 2021 (Gagliardi et al., 2021), this new compilation delves deeper into the realm of digital linguistic biomarkers, shedding light on their evolving relevance and timeliness in the field of psychology and aging.

Our first volume laid the foundation for understanding the potential of digital linguistic biomarkers in assessing various cognitive and psychological aspects. In this second volume, we witness a significant advancement in both the scope and depth of research in this area. The featured articles in this volume contribute to our understanding of how linguistic biomarkers can transcend traditional paper-and-pencil tests, offering a more nuanced and comprehensive approach to the assessment of cognitive function and psychological wellbeing.

In the first study of the volume (Gonzalez-Recober et al.), the authors employed automated methods to investigate speech production during category and letter fluency tasks, commonly used neuropsychological assessments for evaluating lexical retrieval abilities. Their analysis encompassed a diverse range of linguistic and acoustic features, providing a more comprehensive perspective on these tasks than previous studies. As expected, participants produced more words during the category fluency task than during the letter fluency task. Moreover, several linguistic and acoustic measures displayed distinctions between the two tasks. The automated techniques employed in this study offer a reproducible and scalable approach for analyzing fluency tasks, with potential applications in clinical settings. By implementing these methods, future research endeavors are expected to expand our knowledge of speech feature differences, not only in terms of total scores but also across various speech measures, particularly among clinical populations.

In the second article of the volume (Sánchez-Vincitore et al.), the authors present a longitudinal analysis of linguistic biomarkers to detect cognitive decline. Their study underscores the potential of natural language processing techniques in identifying subtle cognitive changes over time. They examined data from over 3,000 participants aged 45 and older to investigate the relationship between age, gender, and language-mediated working memory processes using commercial cognitive tests (in their case, scientific tests developed by CogniFit Inc.). The findings revealed that age negatively predicted working memory performance, highlighting the potential of computerized assessments in predicting cognitive functions during aging and the need for further research on gender effects in cognitive aging. This study contributed to the growing body of evidence supporting the utility of linguistic biomarkers in early cognitive assessment.

In the third study of our volume (Kim et al.), the focus shifts to postoperative delirium (POD) in elderly patients following spinal surgery. POD has been linked to adverse outcomes in this demographic, prompting researchers to explore potential biomarkers for degenerative cerebral dysfunctions like mild cognitive impairment and dementia. The authors used electroencephalography (EEG) to measure an EEG biomarker reflecting idle cortical states through intrinsic alpha oscillations in the prefrontal regions. Cognitive follow-ups were performed using the Telephone Interview for Cognitive Status™ (TICS). The study observed that among patients diagnosed with POD, neurocognitive disorders could persist for up to 1 year post-surgery. These findings suggest that EEG has the potential to be a novel and valuable tool for identifying elderly surgical patients at a higher risk of developing postoperative delirium, offering opportunities for early intervention and improved patient outcomes.

As the fourth article in our volume, the study by Saccone et al. delves into the realm of schizophrenia, examining how it affects speech prosody and pragmatic functions. The study conducted corpus-based research, focusing on real-life spontaneous interactions to shed light on the prosodic features of schizophrenia. Notably, the speech patterns of patients revealed distinct characteristics. Their speech was organized into smaller, less structured information chunks, punctuated by frequent silences and extended pauses during turn-taking. Fluency was disrupted by retracing phenomena, particularly in complex information structures. Besides, comparing Topic and Comment-prominences between patients and non-pathological individuals revealed a consistent pattern. Patients exhibited higher values for Topic-prominence across all parameters, while the non-pathological group displayed the opposite trend. These findings provide valuable insights into the prosodic and pragmatic aspects of speech in schizophrenia, emphasizing the importance of understanding these linguistic manifestations in the context of the disorder's impact on communication.

In closing, the second volume of “*Digital linguistic biomarkers: beyond paper and pencil tests*” presents a short yet diverse and comprehensive array of research articles that collectively advance the field. These contributions not only underscore the relevance and timeliness of linguistic biomarkers in the digital age but also highlight their potential to revolutionize the way we assess cognitive function, psychological wellbeing, and aging across diverse populations, extending to pathological and clinical samples.

## Author contributions

JD: Writing—original draft, Writing—review & editing. DK: Writing—original draft, Writing—review & editing. GG: Writing—original draft, Writing—review & editing.
